# Achieving unlimited recording length in interference lithography via broad-beam scanning exposure with self-referencing alignment

**DOI:** 10.1038/s41598-017-01099-3

**Published:** 2017-04-19

**Authors:** Donghan Ma, Yuxuan Zhao, Lijiang Zeng

**Affiliations:** Department of Precision Instrument, State Key Laboratory of Precision Measurement Technology and Instruments, Tsinghua University, Beijing, 100084 China

## Abstract

Large-area holographic gratings are of great importance in diverse fields including long-range interference metrology, high-resolution astronomical telescopes, and chirped-pulse-amplification systems. However, in conventional interference lithography, the recording length is limited by the aperture of the collimating lenses. Here we propose broad-beam scanning exposure which employs the latent grating generated continuously during scanning for real-time dynamic fringe locking and thus achieves unlimited recording length. This method is experimentally proved to make high-quality gratings, and is expected to be a new type of interference lithography.

## Introduction

Large-area diffraction gratings play an important role in long-range interference metrology^1^, high-resolution astronomical telescopes^2^, and chirped-pulse-amplification systems^3, 4^. In conventional interference lithography, the grating size is limited by the aperture of collimating lenses^5^. Since attaining collimating lenses with large aperture and low aberration is difficult both technologically and financially, it is impractical to make a monolithic large grating in conventional methods. As alternatives, mosaic exposure and scanning exposure have been proposed.

**Figure 1:**
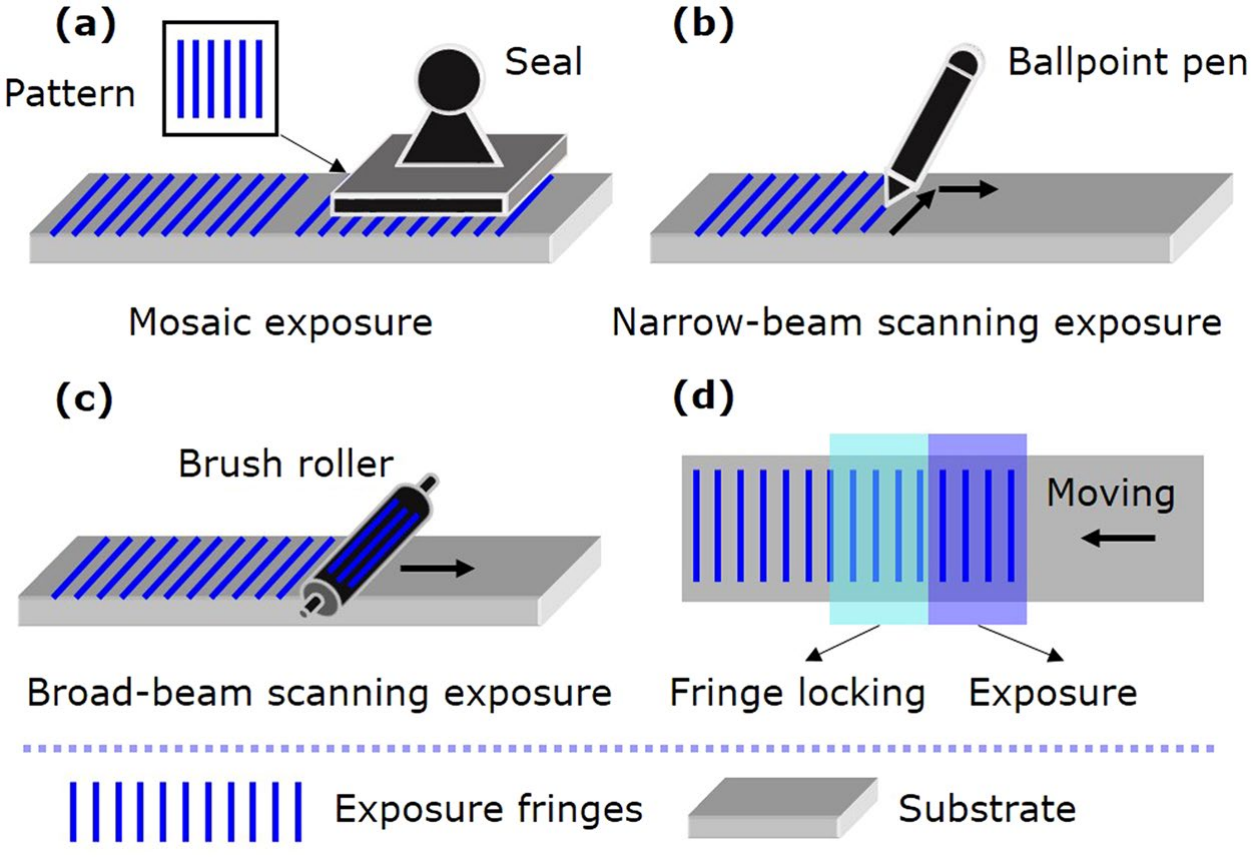
Schematic of different exposure methods. (**a**) Mosaic exposure. (**b**) Narrow-beam scanning exposure. (**c**) Broad-beam scanning exposure. (**d**) Main idea of this work.

In mosaic exposure, a small-size interference field is used as a seal to print the periodic patterns on the large-size substrate step by step, as shown in Fig. 1(a). In this method, one area of the substrate is exposed by the small-size interference field at a time, and then the substrate moves to the next position for the next exposure. In order to keep the wavefront of adjacent exposure areas continuous, it is vital to adjust the phase and attitude of the exposure fringes relative to the substrate between adjacent exposures. In 1996 Turukhano *et al*. proposed a phase synthesis technique to make long grating scales^6^. They utilized narrow auxiliary gratings placed alongside the substrate for phase adjustment and did not consider attitude adjustment. In 2009 Chen *et al*. proposed step-and-align interference lithography to make mosaic gratings on large wafers^7^. They used three red-wavelength interferometers to measure the position and attitude of the substrate and adjusted the translation stage by a three-axis piezo-electric transducer positioned on it. The use of external interferometers separated the alignment and exposure systems, and might introduce drift errors. Recently our group proposed a mosaic technique using diffraction from the latent grating (the exposed but not developed volume grating) for phase and attitude adjustment^8, 9^. The use of the latent grating unified the adjustment and exposure systems, and hence eliminated potential drift errors. However, although mosaic exposure is simple and convenient to make large gratings, the mosaic seam between adjacent exposures degrades the grating quality and brings detrimental effects in use.

Besides mosaic exposure, scanning exposure is another effective method to make large gratings, where the substrate moves continuously through the exposure area. Narrow-beam scanning exposure was proposed by Schattenburg *et al*.^10–15^, where a millimeter-diameter-size interference field was used as a ballpoint pen to write the grating lines on the moving substrate continuously, as shown in Fig. 1(b). The researchers first scanned the substrate along the grating grooves using the millimeter-size interference field. Then they moved the substrate a little along the grating vector (in the direction perpendicular to the grooves) for the next scanning. They measured the position of the substrate by a red-wavelength interferometer and adjusted the phase of the exposure fringes by acoustic optical modulators, to make the fringes “frozen” on the substrate all along during scanning. This system needs sophisticated and costly opto-electronic controls, so it is not easy to be implemented by others. Also, the separation of the measurement and exposure systems may introduce drift errors. Near-field scanning exposure was proposed by Jourlin *et al*.^16–18^, where an expanded laser beam illuminated a transmission grating to generate interference fringes for exposure. They used a grating scale nearby to measure the position of the substrate, and modulated the intensity of the exposure interferogram accordingly. This system is compact and stable, but brings difficulties to suppress the diffraction of unwanted orders^19^. In 2015 we reported our work on broad-beam scanning exposure along the grating vector to make gratings with low stray light^20^. In this method, one half of the interference field was used for exposure, and the other half was used to illuminate the reference grating alongside the substrate for phase and attitude alignment. As a consequence, the recording length was still limited by the size of the reference grating.

Here we develop an improved broad-beam scanning exposure method to achieve unlimited recording length, which takes advantage of the latent grating on the substrate itself for real-time dynamic fringe locking. The use of the latent grating unifies the alignment and exposure systems, and thus makes this method simple and stable. This method has been verified experimentally to make large gratings. Meanwhile, it maintains the advantage of scanning exposure to make gratings with straight grooves, smooth surface, and low stray light^20^. This method is expected to be a new type of interference lithography.

## Results

### Main idea

In this method, broad beam means that the exposure area has a width equal to the substrate width in the grating groove direction and a centimeter-scale length in the grating vector direction. Therefore, the interference field is used as a brush roller to paint the grating structures continuously on the moving substrate from beginning to end, as shown in Fig. 1(c). To avoid fringe smearing during scanning, it is vital to keep the exposure interference fringes stationary relative to the substrate, which is named fringe locking. The latent grating on the exposed area of the substrate can reflect the phase and attitude of the interference fringes relative to the substrate, so it is used for dynamic fringe locking as shown in Fig. 1(d). Since the latent grating is generated continuously on the substrate itself during scanning exposure, this type of interference lithography can achieve unlimited recording length. This is the main idea of our work.

### Scanning exposure system

**Figure 2:**
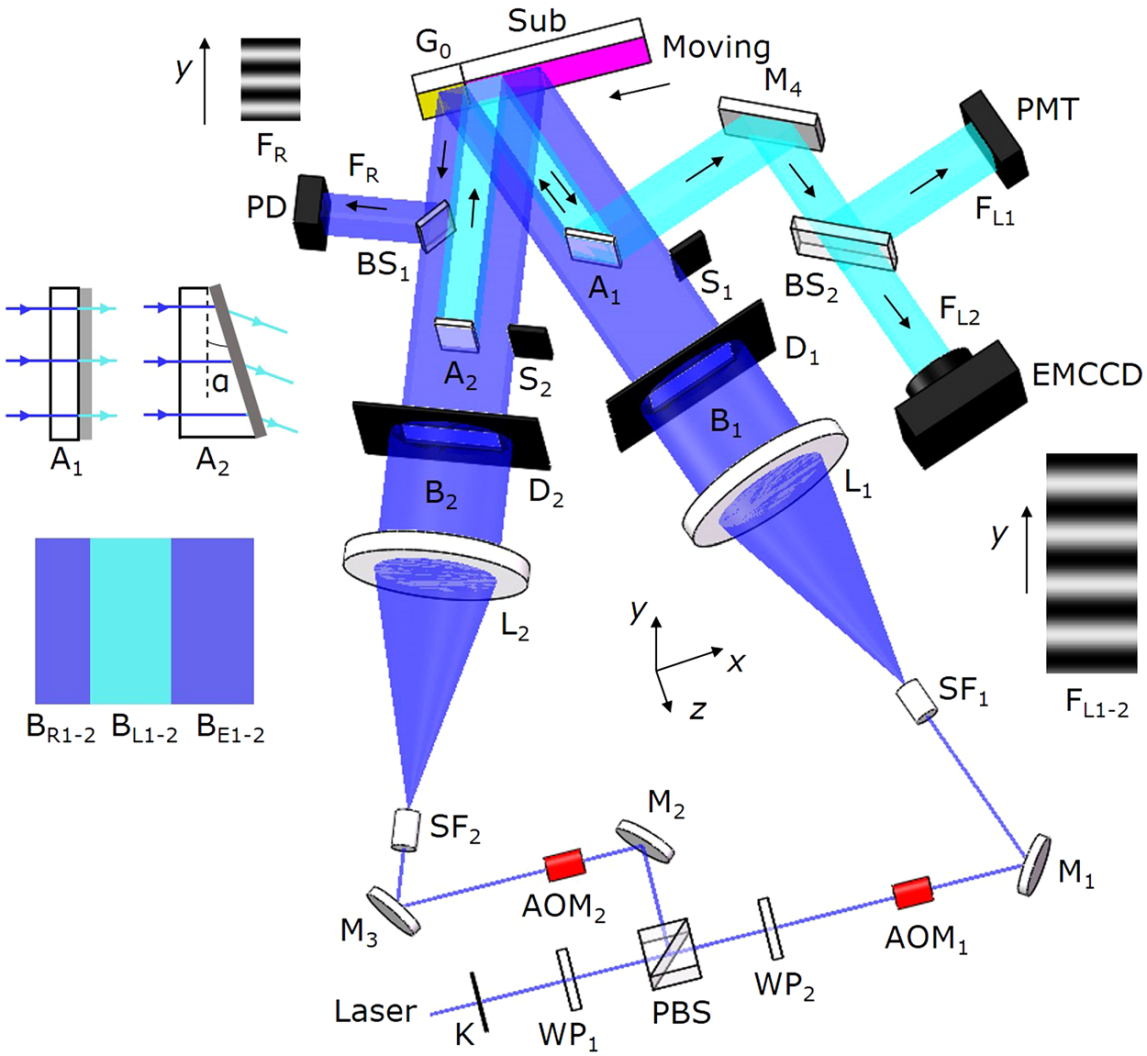
Illustration of experimental setup. K: Shutter; PBS: Polarizing beam splitter; WP_1-2_: Half-wave plate; AOM_1-2_: Acoustic optical modulator; M_1-4_: Mirror; SF_1-2_: Spatial filter; L_1-2_: Collimating lens; D_1-2_: Optical diaphragm; S_1-2_: Baffle; A_1-2_: Attenuator; BS_1-2_: Beam splitter; PD: Photoelectric detector; PMT: Photomultiplier; EMCCD: Electron-multiplying ﻿charge-﻿coupled device; G_0_: Reflective reference grating; Sub: Photoresist-coated substrate; F_R_: Interference fringes generated by the reference grating G_0_; F_L1-2_: Interference fringes generated by the latent grating; B_R1-2_, B_L1-2_, and B_E1-2_: Sub-beams (the subscript R stands for “reference”, L stands for “latent”, and E stands for “exposure”). B_L1_ and B_L2_ are drawn in the lighter color to indicate that their intensities are attenuated by A_1_ and A_2_.

Figure 2 shows the scanning exposure system. The *x*, *y*, and *z* axes are respectively along the directions of grating vector, grating groove, and normal of substrate surface. The photoresist-coated substrate Sub and a prefabricated reference grating G_0_ are mounted abreast with their front surfaces coplanar. They are clamped together on a motorized translation stage (not shown in the figure) that can move along the *x* axis. When the shutter K is open, the laser beam is divided by the polarizing beam splitter (PBS) into two beams. The half-wave plate WP_1_ adjusts their intensity ratio, while WP_2_ makes the transmitted beam have the same polarization state as the reflected one. The two beams are directed by the mirrors M_1_, M_2_, M_3_ and modulated by the acoustic optical modulators AOM_1_ and AOM_2_. The –1st-order diffraction beams from AOM_1_ and AOM_2_ are respectively expanded and cleaned up by the spatial filters SF_1_ and SF_2_, collimated by the lenses L_1_ and L_2_, and become two broad coherent beams B_1_ and B_2_. B_1_ and B_2_ are respectively limited by the optical diaphragms D_1_ and D_2_ to form rectangular cross sections.

Two attenuators A_1_ and A_2_ are used to divide the optical system into an exposure sub-system and a fringe locking sub-system. As shown in Fig. 2, A_1_ is a parallel plate with a transmissivity of 10^−2^, and A_2_ is a plate with a wedge angle *α* = 18″ and a transmissivity of 10^−5^. A_1_ divides the beam B_1_ into three sub-beams named B_R1_, B_L1_, and B_E1_ (the subscript R stands for “reference”, L stands for “latent”, and E stands for “exposure”), while A_2_ divides B_2_ into B_R2_, B_L2_, and B_E2_. The sub-beams B_E1_ and B_E2_ generate interference fringes on the substrate for exposure, while B_R1_, B_R2_, B_L1_, and B_L2_ are used for fringe locking during exposure.

### Fringe locking technique

The simplified steps of scanning exposure are as follows. First move away the attenuators A_1_ and A_2_, and use the sub-beams B_L1_ and B_L2_ to do stationary exposure on an area of the substrate while using the reference grating G_0_ for fringe locking. After that, insert the attenuators into the optical paths and use the latent grating generated on the exposed area for fringe locking. Then move the substrate to pass through the interference field formed by B_E1_ and B_E2_ for scanning exposure. As scanning proceeds, new latent gratings are generated continuously on the newly-exposed area, and they substitute the old ones in subsequent fringe locking.

**Figure 3:**
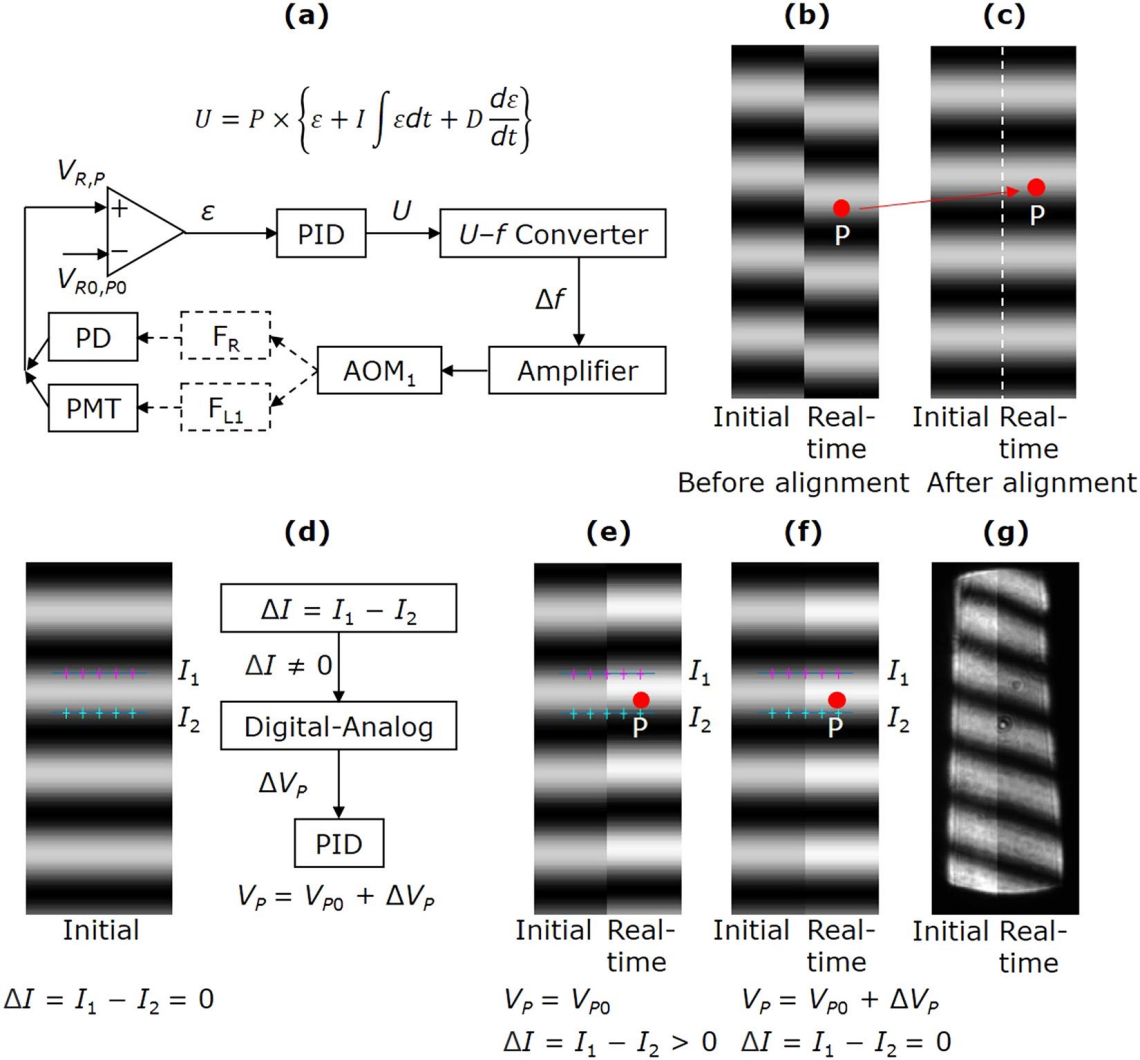
Fringe locking technique. (**a**) Phase locking loop using PD or PMT. (**b**) Phase misalignment between the initial and real-time latent interference fringes when the position of the detection point P is not suitable. (**c**) Phase alignment between the initial and real-time latent interference fringes after adjusting the position of the detection point P along the *y* axis. (**d**) Phase locking loop using EMCCD. (**e**) Phase misalignment when the intensity of the latent interference fringes increases. *V*_*P*_ is locked to *V*_*P*0_, and the real-time average gray level difference Δ*I* > 0. (**f**) Phase alignment after phase locking using EMCCD. *V*_*P*_ = *V*_*P*0_ + Δ*V*_*P*_, and Δ*I* = 0. (**g**) Actual latent interference fringes recorded during scanning exposure. The real-time and initial latent interference fringes are aligned well although the intensity changes obviously.

In stationary exposure, to avoid environmental disturbance, we do phase locking using the reference grating G_0_. As shown in Fig. 2, G_0_ is illuminated by the sub-beams B_R1_ and B_R2_. The 0th order of B_R1_ and the –1st order of B_R2_, diffracted by G_0_, form a set of interference fringes F_R_. F_R_ is reflected by the beam splitter BS_1_ and projected onto the photoelectric detector (PD). We use PD to measure the intensity *V*_*R*_ of one point on F_R_ as the phase information. The difference *ε* between *V*_*R*_ and the target value *V*_*R*0_ is fed to the PID controller, as shown in Fig. 3(a). The output voltage *U* is converted to a frequency signal Δ*f* and amplified to change the frequency of AOM_1_ for phase compensation, until *ε* = 0. In this way, the phase is locked during stationary exposure.

Here we focus on the dynamic fringe locking technique in scanning exposure. As shown in Fig. 2, when the attenuators A_1_ and A_2_ are inserted into the optical paths, the latent grating is illuminated by the attenuated sub-beams B_L1_ and B_L2_. The + 1st order of B_L1_ and the 0th order of B_L2_, diffracted by the latent grating, form a set of interference fringes, which is named latent interference fringes. The latent interference fringes are reflected by A_1_ and M_4_, and divided into F_L1_ and F_L2_ by the beam splitter BS_2_. F_L1_ is projected onto the photomultiplier (PMT), while F_L2_ is recorded by the electron-multiplying ﻿charge-﻿coupled ﻿device (EMCCD). We use the latent interference fringes F_L1_ and F_L2_ together for phase and attitude locking.

In order to get high response frequency and low intensity dependence, we utilize PMT and EMCCD together for phase locking. When the substrate moves, to avoid fringe smearing, the scanning formula Δ*f* = *v*/*d* ^10^ should be strictly satisfied, where Δ*f* is the frequency difference between the two beams, *v* is the moving speed of the substrate, and *d* is the period of the exposure interference fringes. If the scanning formula is not satisfied, the latent interference fringes will have a phase shift. Similarly, we use PMT to measure the real-time intensity *V*_*P*_ of the detection point P on F_L1_, get the error *ε* between *V*_*P*_ and the target value *V*_*P*0_, and then feed it back to AOM_1_ for phase compensation, as shown in Fig. 3(a).

Two points should be noted when using PMT for phase locking. First, the target value *V*_*P*0_ of phase locking will affect the locking sensitivity and stability. Second, after phase locking, the real-time latent interference fringes should be aligned with the initial ones to keep the wavefront continuous between the scanning and stationary exposure areas.

First we choose a suitable target value *V*_*P*0_. If the scanning formula Δ*f* = *v*/*d* is not satisfied, the intensity *V*_*P*_ varies as a sinusoidal curve and has the largest derivative in the mid-value of its range. We set Δ*f* = 1 Hz before locking to find the maximum and minimum values of *V*_*P*_, and then set *V*_*P*0_ = (*V*_*P*max_ + *V*_*P*min_)/2. In this case, locking *V*_*P*_ to *V*_*P*0_ is sensitive and stable.

Then we align the fringes. Right after stationary exposure, we use EMCCD to record the initial latent interference fringes F_L2_, and then lock *V*_*P*_ to *V*_*P*0_ as mentioned above. At this time, the real-time and initial latent interference fringes are usually misaligned as shown in Fig. 3(b). We move PMT along the *y* axis to adjust the position of the detection point P while comparing the real-time and initial latent interference fringes from EMCCD, until they are aligned as shown in Fig. 3(c). In this case, we can realize good alignment as well as sensitive and stable locking.

In another aspect, since this phase locking is based on the intensity of one single point, it is easy to be affected by the intensity variation of the latent interference fringes, which is inevitable due to the non-uniformity of the photoresist thickness. In order to eliminate the influence of the intensity variation, we use EMCCD to adjust the target value *V*_*P*0_. An image-processing program is used to extract the phase information from the latent interference fringes F_L2_. The program samples two rows of points that are symmetrical about one arbitrary fringe in the initial image, as shown in Fig. 3(d). Then the program calculates the average gray level *I*_1_ of the pink points and *I*_2_ of the blue points. Because of symmetry, the average gray level difference Δ*I* = *I*_1_ − *I*_2_ = 0. Moreover, as long as the phase does not shift, Δ*I* will be zero no matter how the average intensity changes. During scanning exposure, when the intensity of the latent interference fringes changes, for example increases as shown in Fig. 3(e), if *V*_*P*_ is still locked to *V*_*P*0_, the real-time fringes will shift up, resulting in Δ*I* = *I*_1_ − *I*_2_ > 0. Then the program outputs a voltage Δ*V*_*P*_ to the PID controller, which locks *V*_*P*_ to *V*_*P*0_ + Δ*V*_*P*_ and thus pulls the fringes back. The locking loop repeats until Δ*I* = 0, as shown in Fig. 3(f). Figure 3(g) shows the actual latent interference fringes recorded during scanning exposure. It is seen that although the average intensity of the fringes varies obviously, the phases of the real-time and initial fringes are still aligned, which verifies the intensity independence of phase locking.

Besides the phase error, the angular errors of the translation stage lead to the attitude errors of the substrate relative to the exposure fringes. The rotation of the substrate about the *z* axis (the pitch angle of the stage) gives rise to an error in groove direction, meaning a spacing variation of the latent interference fringes. It can be compensated by shifting the pinhole of SF_2_ along the *y* axis. The rotation of the substrate about the *y* axis (the yaw angle of the stage) leads to an error in grating period, meaning a tilt of the latent interference fringes. It can be compensated by shifting the pinhole of SF_2_ in the direction perpendicular to the beam in the *x*–*z* plane. Since this error is second-order of the small rotation angle, it is omitted in experiments. The movements of the substrate along the *y* and *z* axes and the rotation about the *x* axis have no influence on exposure. The attitude errors are also extracted from the latent fringe images and compensated by adjusting the exposure fringes accordingly. Since the algorithm is similar to phase locking, here we will not give more details.

From above it is seen that the dynamic fringe locking technique has high locking stability and low intensity dependence. With this technique, the latent interference fringes are kept stationary during scanning exposure. In other words, the exposure fringes are “frozen” on the substrate.

### Linear exposure technique

Linear exposure is another key technique in order to make the exposure dose uniform. When talking about the exposure doses, we do not consider the non-uniformity of the beams themselves.

**Figure 4:**
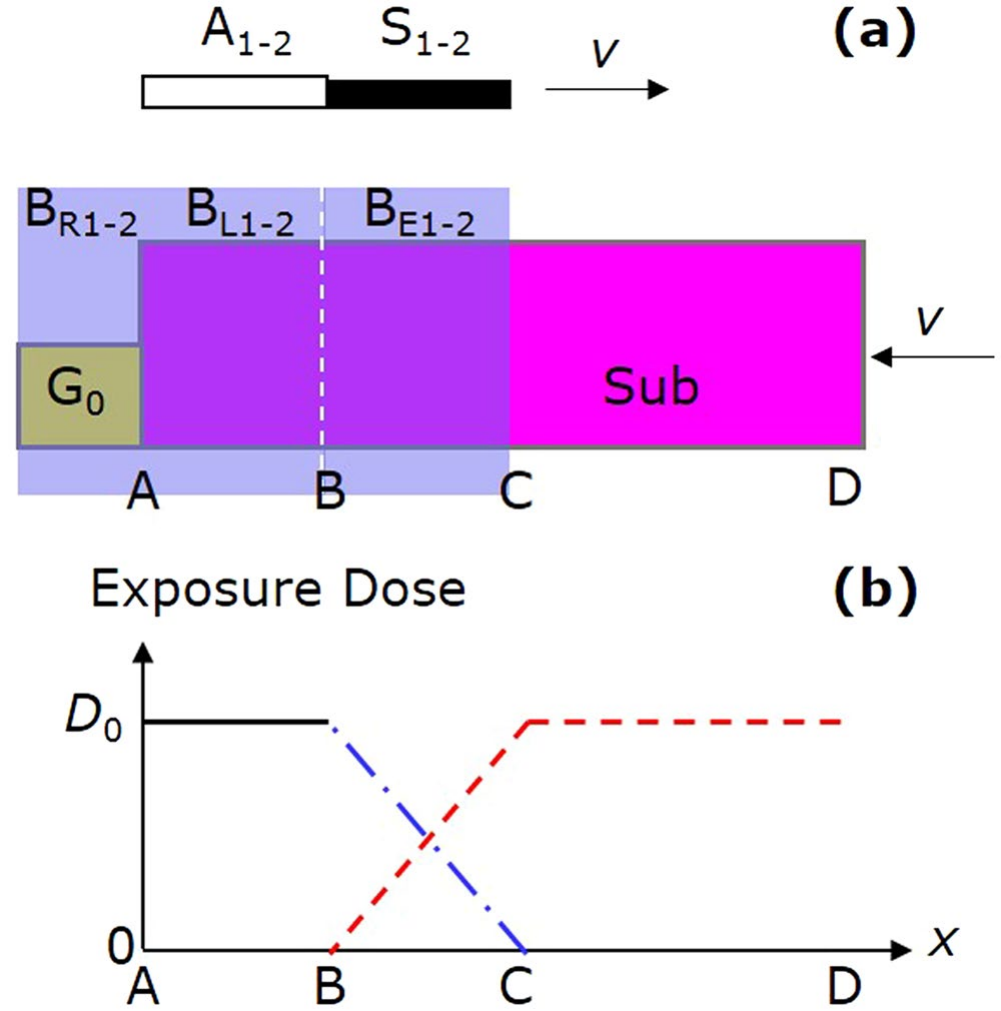
Linear exposure technique. (**a**) Exposure procedure. The pink and yellow rectangles denote the substrate and the reference grating G_0_ respectively. The blue rectangles denote the sub-beams B_R1-2_, B_L1-2_, and B_E1-2_. The gray rectangle denotes the attenuators A_1-2_. The black rectangle denotes the baffles S_1-2_. (**b**) Exposure dose distribution. The black solid, blue dash-dotted, and red dashed lines respectively denote the exposure doses during stationary, linear, and scanning exposures.

As shown in Fig. 4(a), before exposure the baffles S_1_ and S_2_ block B_E1_ and B_E2_ respectively, and the attenuators A_1_ and A_2_ are not in the optical paths. When exposure starts, we move S_1_ and S_2_ along the + *x* direction, and use B_L1_, B_L2_ and the unblocked parts of B_E1_ and B_E2_ to expose the areas AB and BC. The moving speed of the baffles satisfies *v* = *b*_*E*_/*T*, where *b*_*E*_ is the length of B_E1-2_, and *T* is the exposure time. As shown in Fig. 4(b), when this exposure ends, the exposure dose of the area AB is a constant *D*_0_ as the black solid line shows, while that of the area BC changes from *D*_0_ to 0 linearly as the blue dash-dotted line shows. Then we insert A_1_ and A_2_ into the optical paths to get the latent interference fringes. After that, we start to move the substrate in a speed *v* and use B_E1_ and B_E2_ for scanning exposure. During scanning exposure, the exposure doses of the areas BC and CD are shown as the red dashed lines in Fig. 4(b). Therefore, the total exposure doses of the areas AB, BC, and CD are all equal to *D*_0_. In this way, we can make gratings with a uniform exposure dose along the grating vector direction. Moreover, using this exposure procedure can avoid the mosaic seam between the areas AB and BC. We refer to the areas AB, BC, and CD as the stationary, linear, and scanning exposure areas.

**Figure 5:**
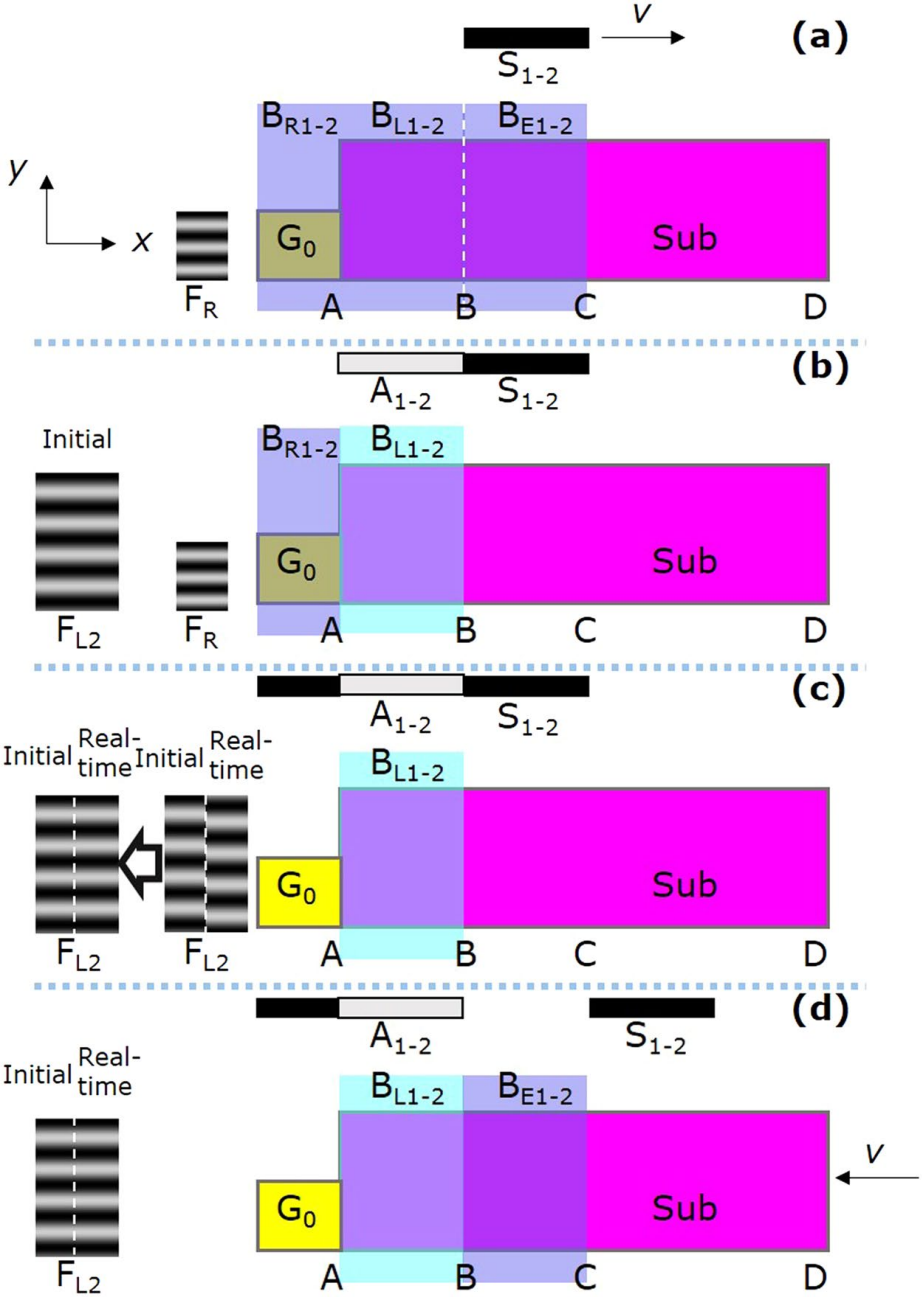
Scanning exposure procedure. (**a**) Stationary and linear exposures. (**b**) Initial latent interference fringes recording. (**c**) Dynamic fringe locking preparation. (**d**) Scanning exposure.

The scanning exposure procedure is shown in Fig. 5. After stationary and linear exposures [Fig. 5(a)] we get the initial latent interference fringes, and record them using EMCCD [Fig. 5(b)]. Then we prepare for the dynamic fringe locking [Fig. 5(c)] and start scanning exposure [Fig. 5(d)], until the whole substrate moves out of the exposure interference field. More details can be found in Methods.

### Experimental results

To prove the feasibility of this exposure method, we made gratings with different photoresist thickness and duty cycles (the ratio of the linewidth to the grating period). Gratings were fabricated on quartz substrates of a size 200 mm × 100 mm. A chromium film of about 120 nm thick was coated on the substrates. The grating period *d* = 570 nm, which was determined by the exposure wavelength *λ*_*E*_ and the half-angle *θ* between the beams B_1_ and B_2_ as *d* = *λ*_*E*_/(2sin*θ*). The duty cycle was determined by the exposure dose and the development time^21^.

**Figure 6:**
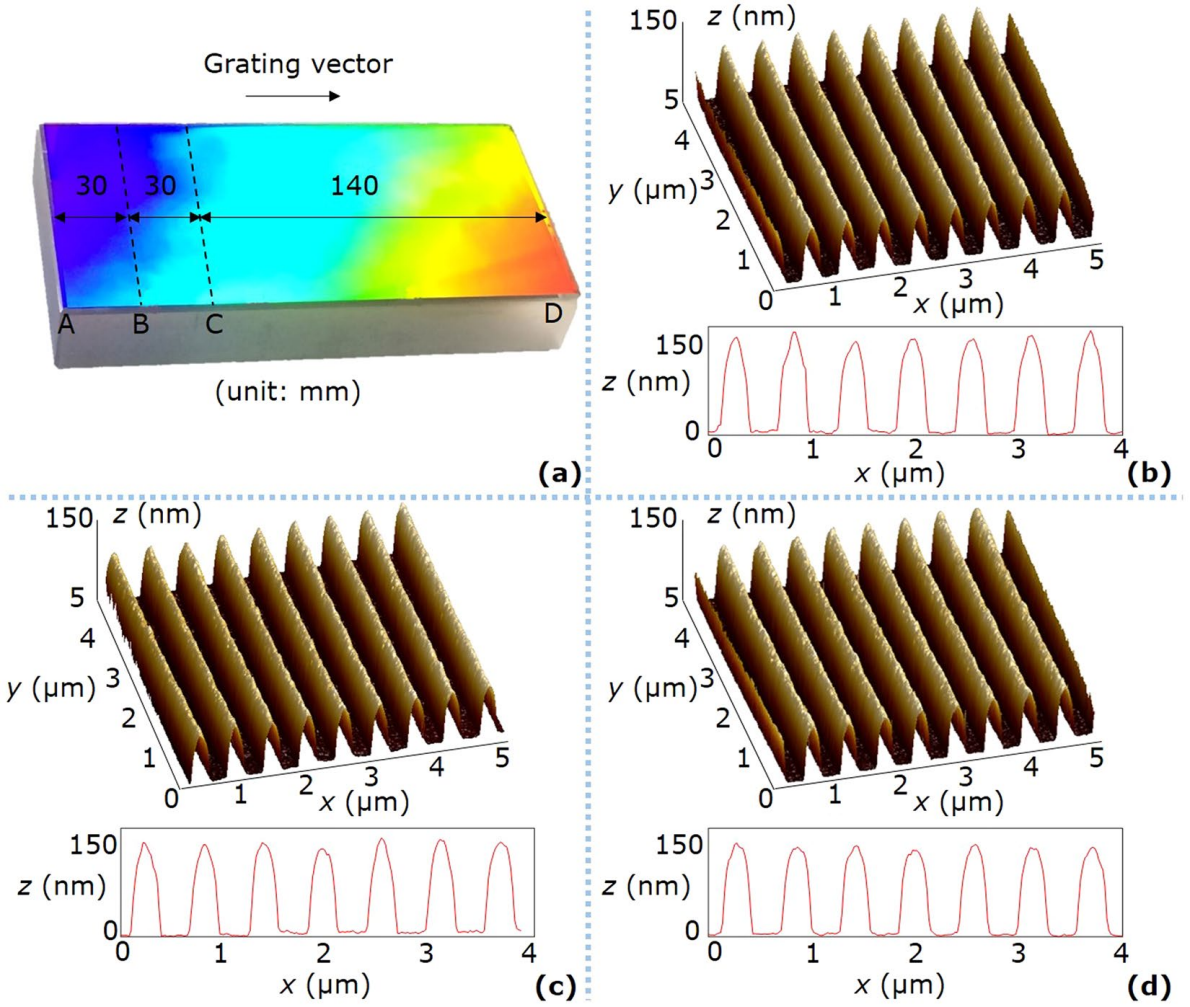
Photograph and AFM images of the grating with a photoresist thickness of 150 nm. (**a**) Photograph of the grating. AB is the stationary exposure area of a size 30 mm × 100 mm, BC is the linear exposure area of a size 30 mm × 100 mm, and CD is the scanning exposure area of a size 140 mm × 100 mm. (**b**) AFM image of the sample point on the area AB. The average (AVE) and standard deviation (STD) of the duty cycles are respectively 0.41 and 0.02. (**c**) AFM image of the sample point on the area BC. The AVE and STD of the duty cycles are respectively 0.40 and 0.02. (**d**) AFM image of the sample point on the area CD. The AVE and STD of the duty cycles are respectively 0.41 and 0.01. For (**b**–**d**), the measurement area is 5 μm × 5 μm, and the groove structures in the mid-line of the measurement area are shown at the bottom of each sub-figure.

**Figure 7:**
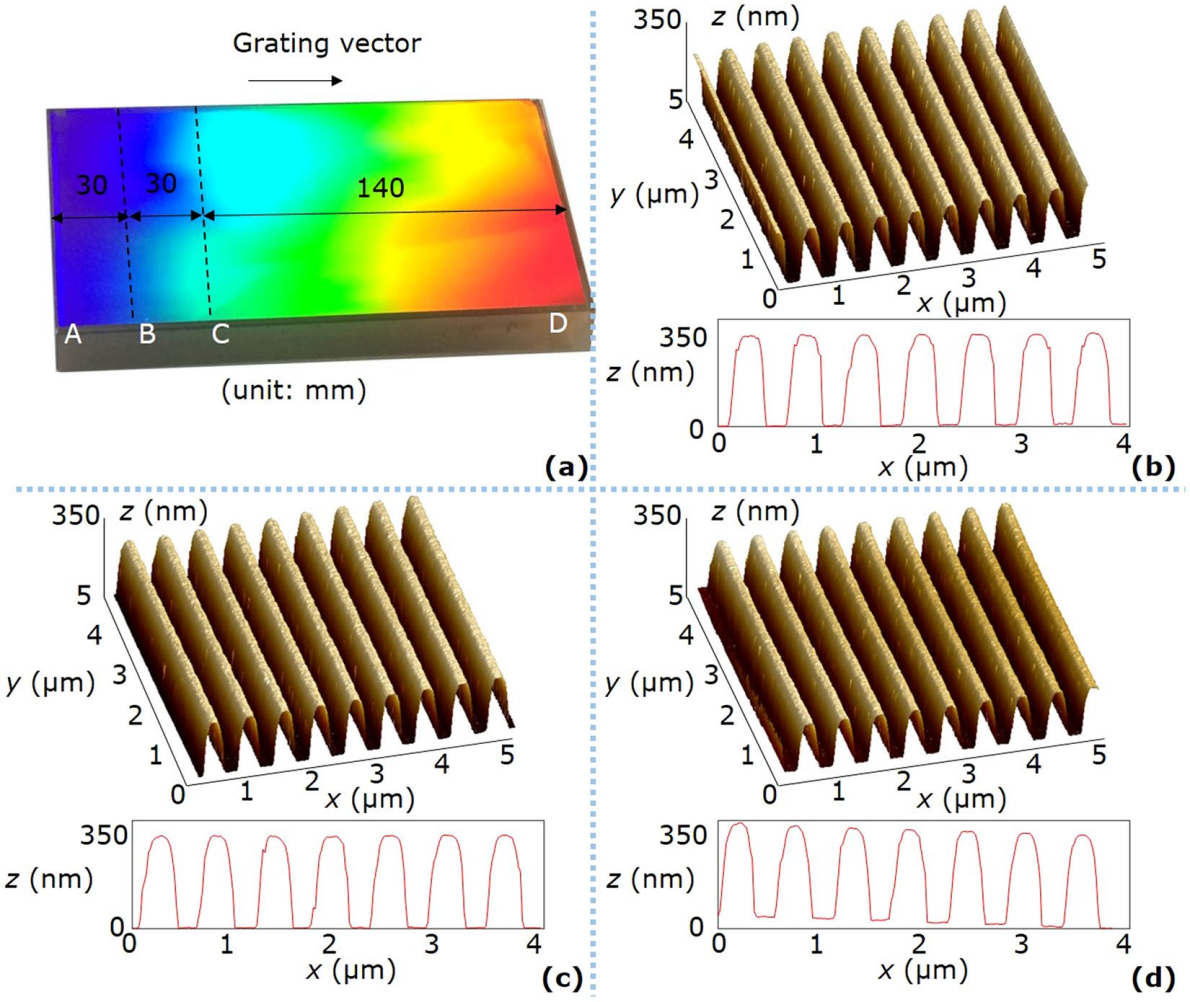
Photograph and AFM images of the grating with a photoresist thickness of 340 nm. (**a**) Photograph of the grating. AB is the stationary exposure area of a size 30 mm × 100 mm, BC is the linear exposure area of a size 30 mm × 100 mm, and CD is the scanning exposure area of a size 140 mm × 100 mm. (**b**) AFM image of the sample point on the area AB. The AVE and STD of the duty cycles are respectively 0.52 and 0.01. (**c**) AFM image of the sample point on the area BC. The AVE and STD of the duty cycles are respectively 0.53 and 0.01. (**d**) AFM image of the sample point on the area CD. The AVE and STD of the duty cycles are respectively 0.51 and 0.01. For (**b**–**d**), the measurement area is 5 μm × 5 μm, and the groove structures in the mid-line of the measurement area are shown at the bottom of each sub-figure.

Figure 6(a) shows the photograph of a grating with a photoresist thickness of 150 nm. The whole grating is bright and uniform by appearance. We randomly chose three sample points on different exposure areas and observed their microstructures by the atomic force microscope (AFM), as shown in Fig. 6(b–d). The average duty cycles of the stationary, linear, and scanning exposure areas are respectively 0.41, 0.40, and 0.41 in the measurement area of 5 μm × 5 μm. Similarly, the photograph of a grating with a photoresist thickness of 340 nm is shown in Fig. 7(a), and the microstructures of the randomly-chosen sample points are shown in Fig. 7(b–d). The average duty cycles of the stationary, linear, and scanning exposure areas are respectively 0.52, 0.53, and 0.51 in the measurement area of 5 μm × 5 μm. Therefore, from both macro and micro perspectives, the grating structures in the stationary, linear, and scanning exposure areas do not have obvious difference, proving that the fabricated gratings have good uniformity.

**Figure 8:**
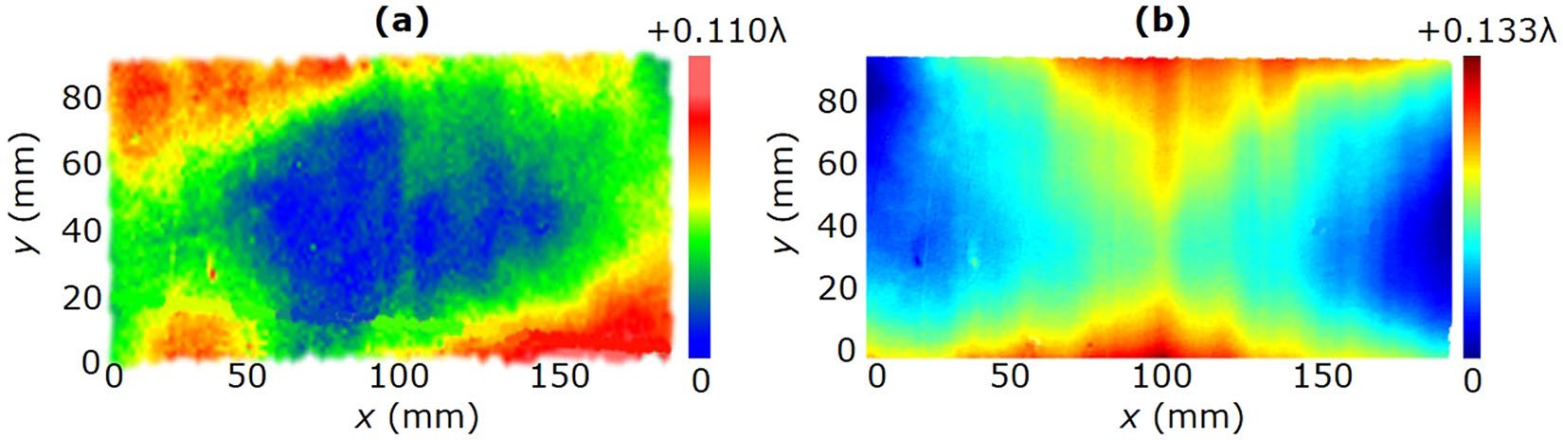
Wavefront measurement results. (**a**) Non-flatness of the substrate. The PV and RMS values are respectively 0.110 λ and 0.021 λ. (**b**) Spacing error of the grating. The PV and RMS values are respectively 0.133 λ and 0.023 λ. For both (**a**,**b**), the measurement area is 187 mm × 87 mm.

We used the Zygo interferometer to measure the non-flatness of the substrate and the spacing error^22^ of the grating in Fig. 6. The measurement area is 187 mm × 87 mm. The non-flatness distribution of the substrate is shown in Fig. 8(a), whose peak-valley (PV) and root-mean-square (RMS) values are 0.110 λ and 0.021 λ, respectively. The spacing error distribution of the grating is shown in Fig. 8(b), whose PV and RMS values are 0.133 λ and 0.023 λ, respectively. These wavefront measurement results show the high quality of the fabricated grating.

Above-mentioned experimental results prove the feasibility of this method in fabricating large-area gratings.

## Discussion

First, we analyze the fringe locking frequency and resolution. The bandwidth of the PID controller is 100 kHz. Due to the electronic noise, the intensity signal fluctuation is 100 mV. In experiments, we have *V*_*P*max_ = 2.0 V, *V*_*P*min_ = 0.6 V, and the target value *V*_*P*0_ = (*V*_*P*max_ + *V*_*P*min_)/2 = 1.3 V. So the signal fluctuation of 100 mV means a phase variation Δ*φ* = 100 × 10^−3^ × 2/(2.0 − 0.6) = 0.045π. Therefore, the phase is locked to *V*_*P*0_ with a resolution of 0.045π every 10 μs. The refresh frequency of Δ*V*_*P*_ is 1 Hz. The latent interference fringes recorded by EMCCD have a spacing of 30 pixels, while the image algorithm can recognize the mismatch of one pixel. Therefore, the target value is finely adjusted at a frequency of 1 Hz with a resolution of 1/30 × 2π = 0.067π. The refresh frequency of attitude locking is 20 Hz. The recorded latent interference fringes have a height of 200 pixels. When the substrate is rotated Δ*θ*_*z*_ about the *z* axis, the spacing variation equals Δ*e* = *e*_0_^2^Δ*θ*_*z*_/*d*, where the fringe spacing *e*_0_ = 10 mm, Δ*e*/*e*_0_ = 1/200, and *d* = 570 nm. Therefore, the attitude is aligned at a frequency of 20 Hz with a resolution of Δ*θ*_*z*_ = 1/200/10 × 570 × 10^−6^ = 0.3 μrad = 0.06″.

Second, we remark on the applicable range of our method regarding the grating period. The theoretical minimal grating period satisfies *d* = *λ*_*E*_/2 = 199 nm, when the half-angle *θ* between the beams B_1_ and B_2_ equals 90°. However, to make such a small-period grating, the scanning exposure system demands higher stability, thus we may need to increase the fringe locking frequency and accuracy. The maximal grating period is limited only by the spatial layout constraint of the exposure system. The need for a very small half-angle *θ* is in conflict with the desirable aperture size of the collimating lenses and the available length of the optical table.

Third, we compare the broad-beam scanning exposure with the previous methods. Compared with the mosaic exposure method, this method eliminates the seams of mosaic gratings which have detrimental effects in grating applications. Compared with previous scanning exposure methods, this method employs the latent grating for dynamic fringe locking, which unifies the exposure and alignment systems and does not need sophisticated controls.

It is necessary to note that although we only made gratings of a size 200 mm × 100 mm in experiments, this method has no recording length limit in principle due to the use of the latent grating. Its recording length only depends on the length of the substrate.

In conclusion, we develop a broad-beam scanning exposure method which eliminates the recording length limit in conventional interference lithography. It takes advantage of the latent grating to adjust the phase and attitude of the exposure fringes relative to the substrate in real time, so it is a self-referencing method. Its feasibility in grating fabrication has been proved experimentally. Therefore, this method is simple and effective, and is expected to be a new type of interference lithography.

## Methods

### Scanning exposure procedure

The detailed scanning exposure procedure is as follows.

1. Do stationary and linear exposures.
  Do stationary exposure using the unattenuated sub-beams B_L1-2_ for a time *T*. The phase locking is performed with F_R_. As soon as the exposure starts, move the baffles S_1-2_ along the +*x* direction in a speed *v* = *b*_*E*_/*T* to do linear exposure using B_E1-2_.
2. Record the initial latent interference fringes.
  Insert S_1-2_ again to block B_E1-2_, and insert the attenuators A_1-2_ to generate latent interference fringes. Continue phase locking using F_R_ and record the initial latent interference fringes F_L2_ by EMCCD. Use the image-processing program to extract the phase and attitude information from the initial image.
3. Prepare for dynamic fringe locking.
  Stop phase locking using F_R_ and block B_R1-2_. Set Δ*f* = 1 Hz to obtain the reading range of *V*_*P*_ from PMT, and set *V*_*P*0_ to be the mid-value of the range. Lock *V*_*P*_ to *V*_*P*0_ using the PID controller, and then move PMT along the *y* axis while comparing the real-time and initial latent interference fringes from EMCCD, until they are aligned.
4. Do scanning exposure.
  Move away S_1-2_. Move the substrate and G_0_ together along the –*x* direction in a speed *v* for scanning exposure while locking the latent interference fringes using PMT and EMCCD. Exposure ends until the entire substrate moves out of the illumination of B_E1-2_.
5. Development.

﻿﻿After development we obtain a grating of a long size along the *x* axis.

### Experimental parameters

The exposure light source was a semiconductor laser of a wavelength 397.5 nm, a power 600 mW, and a coherence length longer than 100 m. The collimating lenses were biconvex spherical lenses of a focus length 1080 mm and an aperture diameter 180 mm. The length *b*_*R*_ of B_R1-2_ was 20 mm. The length *b*_*L*_ of B_L1-2_ was 30 mm. The length *b*_*E*_ of B_E1-2_ was 30 mm. The translation stage had a travel range of 350 mm along the *x* axis with a pitch angle of 6″ and a yaw angle of 2″ for the whole range. The nano-positioning stage, on which SF_2_ was mounted, had a travel range of 20 μm along the *y* axis. The reference grating G_0_ was prefabricated by stationary exposure in this system, with a usable area of 20 mm × 20 mm.

Positive photoresist Shipley 9912 was used. For the grating with a photoresist thickness of 150 nm, the exposure time *T* was 80 s, and the scanning speed *v* = *b*_*E*_/*T* = 0.375 mm/s. For the grating with a photoresist thickness of 340 nm, *T* was 200 s, and *v* was 0.150 mm/s.
